# Inflammatory Cytokines in General and Central Obesity and Modulating Effects of Physical Activity

**DOI:** 10.1371/journal.pone.0121971

**Published:** 2015-03-17

**Authors:** Frank M. Schmidt, Julia Weschenfelder, Christian Sander, Juliane Minkwitz, Julia Thormann, Tobias Chittka, Roland Mergl, Kenneth C. Kirkby, Mathias Faßhauer, Michael Stumvoll, Lesca M. Holdt, Daniel Teupser, Ulrich Hegerl, Hubertus Himmerich

**Affiliations:** 1 Leipzig University Medical Center, Integrated Research and Treatment Center Adiposity Diseases (IFB), Leipzig, Saxony, Germany; 2 Department of Psychiatry and Psychotherapy, University Hospital Leipzig, Leipzig, Saxony, Germany; 3 Department of Psychiatry, University of Tasmania, Hobart, Tasmania, Australia; 4 Institute of Laboratory Medicine, Ludwig-Maximilians-University Munich, Munich, Bavaria, Germany; GDC, GERMANY

## Abstract

**Context:**

Chronic systemic inflammation in obesity originates from local immune responses in visceral adipose tissue. However, assessment of a broad range of inflammation-mediating cytokines and their relationship to physical activity and adipometrics has scarcely been reported to date.

**Objective:**

To characterize the profile of a broad range of pro- and anti-inflammatory cytokines and the impact of physical activity and energy expenditure in individuals with general obesity, central obesity, and non-obese subjects.

**Design, Setting, and Participants:**

A cross-sectional study comprising 117 obese patients (body mass index (BMI) ≥ 30) and 83 non-obese community-based volunteers.

**Main Outcomes Measures:**

Serum levels of interleukin (IL)-2, IL-4, IL-5, IL-10, IL-12, IL-13, granulocyte-macrophage colony-stimulating factor (GM-CSF), interferon (IFN)-γ and tumor necrosis factor (TNF)-α were measured. Physical activity and energy expenditure (MET) were assessed with actigraphy. Adipometrics comprised BMI, weight, abdominal-, waist- and hip-circumference, waist to hip ratio (WHR), and waist-to-height-ratio (WHtR).

**Results:**

General obesity was associated with significantly elevated levels of IL-5, IL-10, IL-12, IL-13, IFN-γ and TNF-α, central obesity with significantly elevated IL-5, IL-10, IL-12, IL-13 and IFN-γ-levels. In participants with general obesity, levels of IL-4, IL-10 and IL-13 were significantly elevated in participants with low physical activity, even when controlled for BMI which was negatively associated with physical acitivity. Cytokines significantly correlated with adipometrics, particularly in obese participants.

**Conclusions:**

Results confirm up-regulation of certain pro- and anti-inflammatory cytokines in obesity. In obese subjects, physical activity may lower levels and thus reduce pro-inflammatory effects of cytokines that may link obesity, insulin resistance and diabetes.

## Introduction

Obesity is a medical condition characterized by excessive body fat with a body mass index (BMI) exceeding 30 kg/m^2^, which leads to serious impairment of health [1]. With more than 500 million people worldwide currently affected, obesity and highly co-morbid disorders like metabolic syndrome (MetS), cardiovascular diseases, diabetes, sleep disorders and chronic inflammatory diseases present major health concerns in developed and developing countries [1, 2]. Former interpretations of obesity as a life-style issue simply resulting from an imbalance between energy intake and expenditure have given way to evidence of more complex and multifactorial pathogenic processes. Adipose tissue (AT) is not only an energy reservoir but a multifunctional endocrine organ secreting a range of bioactive peptides and proteins [3]. These adipocyte-derived adipokines are a heterogeneous group including cytokines, hormones, growth factors, acute phase proteins, prostaglandins, glucocorticoids and sex steroids, with complex effects on the receptor organs liver, pancreas, skeletal muscle, kidneys, hypothalamus and the immune system [4]. In obesity, alterations of adipokines and several further cytokines are thought to contribute to a low grade inflammation within the AT affecting the development of several secondary diseases such as MetS, insulin resistance (IR), diabetes, arterial hypertension and asthma [5–7].

Changes in cytokine release are related to the infiltration of macrophages into AT that follow the adipocyte-secretion of chemoattractants like tumor-necrosis-factor alpha (TNF-α) and free fatty acids [8]. The shift in the activation state of macrophages from mainly alternatively activated (M2) to classically activated macrophages (M1) is enhanced in obesity and controlled by a number of cytokines. Thus, interleukin (IL)-13, and IL-4 show mainly pro-M2-properties [9, 10], whereas interferon (IFN)-γ and granulocyte macrophage colony-stimulating factor (GM-CSF) exhibit pro-M1-properties [11, 12]. Closely related to macrophage polarization is the shift from T-helper cells 2 (T_H_2) to T-helper cells 1 (T_H_1) and altered activity of regulatory T (T_reg_) cells in obesity [10]. In obesity, under a high-fat diet (HFD), pro-inflammatory T_H_1 and M1 macrophages were reported to be activated and to produce IFN-γ, TNF-α, and IL-12 [11, 13], whereas the differentiation of naïve T-cells into anti-inflammatory T_H_2 which secrete IL-4, IL-10 and IL-13, as well as the activity of T_reg_ cells, were reduced [14].

To date, in-vivo serum studies with respect to levels of serum cytokines in subjects suffering from obesity and MetS are scarce. The secretion of pro-inflammatory adipokines by hypertrophied adipocytes of visceral AT, predominately TNF-α and IL-6, has been reported increased in obese subjects [3, 15], whereas the secretion of anti-inflammatory adipokines seems to be suppressed [16]. Levels of IL-12 were elevated in obesity [17], IFN-γ, IL-4, IL-5, IL-12 and IL-13 elevated in MetS [18]. Inconsistent results were found for IL-10 levels [16, 19]. In obese adolescent girls, TNF-α, IL-4 and IL-5 levels were higher in those with central obesity [20]. For several cytokines it has been reported that their concentrations correlate with BMI [21, 22]. Thus, by reducing BMI and especially visceral AT, physical exercise is able to reduce the production and circulation of pro-inflammatory mediators [23, 24].

In order to investigate the role of a broad range of pro- and anti-inflammatory cytokines in obesity, serum levels of TNF-α, IFN-γ, IL-2, IL-4, IL-5, IL-10, IL-12, IL-13, and GM-CSF were compared between obese and non-obese participants. Since physical activity has been shown to modulate weight and levels of pro-inflammatory mediators [23, 25], cytokine levels were further combined with actigraphic assessment of activity including the amount of steps and the determination of the ‘Metabolic Equivalent of Task (MET)’. A MET is a physiological measure of the energy costs of physical activity and is defined as the ratio of metabolic rate during a physical activity to a reference metabolic rate. To further elucidate the role of fat distribution, cytokines were compared between participants with and without central obesity and correlated with a range of adipometrics.

To our knowledge, this is the first study investigating IL-2, IL-13 and GM-CSF with regard to central obesity and the first combining cytokine levels and objective actigraphic measures of physical activity in obese participants. We hypothesized pro-inflammatory cytokines to be elevated in obese compared to non-obese participants and in participants with central obesity compared to those without central obesity. We further hypothesized levels of pro-inflammatory cytokines to be elevated in participants with low vs. high physical activity within the separate groups.

## Materials and Methods

### Participants

We recruited 240 participants of which 200 were included into final analyses: 117 obese patients (BMI ≥ 30) from the outpatient clinic of the Integrated Research and Treatment Center for Adiposity Diseases Leipzig (IFB) and a control group of 83 non-obese (BMI < 30) volunteers recruited via announcements (intranet, internet, local newspapers). Without reference to their BMI category, all 200 participants were separately categorized into with central obesity (n = 69) and without central obesity (n = 131) groups, according to the waist-to-hip-ratio (WHR; central obesity ≥ 0.85 in females, ≥ 0.90 in males). Thus, the without central obesity group included the 15 participants from the obesity group with only general but not central obesity. Evaluation of inclusion and exclusion criteria for the study was performed in two stages. First, potential participants were contacted via phone and invited to participate in a telephone screening interview, comprising socio-demographic data, screening for somatic disorders and a checklist of the Structured Clinical Interview for DSM-IV [26]. Eligible participants were then invited to the study centre, where exclusion criteria were assessed in more detail. Exclusion criteria were acute or chronic infections, current medication with a recognized impact on the immune system, psychiatric and neurological disorders, and a history of head injury with loss of consciousness exceeding 1 hour. Assessments for current and past history of physical and mental health problems as well as current medication were performed using standardized forms. We also assessed co-morbidities and current medications (Table 1). All participants were aged 18 to 70 years. Written informed consent was obtained from all participants. The study was approved by Leipzig University Ethics Committee (#015–10–18012009).

**Table 1:** Comorbidities and current medication in obese and non-obese participants.

	Obese (n = 117)	Non-obese (n = 83)	p value (Fisher's exact test)
Comorbidities			
**Hypertension**	55	3	< 0.001
**Arthrosis**	35	0	NA
**Diabetes mellitus**	28	0	NA
**Hypothyreosis**	24	4	= 0.002
**Asthma**	15	3	= 0.026
**Cardiovascular disease**	12	0	NA
**Sleep apnea**	7	0	NA
**Steatosis hepatis**	6	0	NA
**Gastroesophageal reflux disease**	6	0	NA
**Atopic disease**	5	2	= 0.702
**Glaucoma**	4	0	NA
**Articular gout**	2	0	NA
**Polycystic ovary syndrome**	1	0	NA
Current medication			
**Antihypertensives**	50	3	< 0.001
Beta blockers	18	2	= 0.003
ACE Inhibitoren	20	1	< 0.001
Diuretics	25	0	NA
Calcium antagonists	5	0	NA
AT1 antagonists	13	0	NA
Alpha2 agonist	3	0	NA
Alpha1 blocker	2	0	NA
**Oral antidiabetics**	24	0	NA
**Thyroid hormones**	16	4	= 0.052
**Contraceptives**	11	9	= 0.813
**Uricostatics**	10	0	NA
**NSAID**	8	0	NA
**Insulin**	7	0	NA
**Proton pump inhibitors**	6	0	NA
**Statins**	4	0	NA
**Bronchodilators**	2	1	= 1.000
**Nitrates**	2	0	NA
**Glucocorticoids**	1	1	= 1.000
**Vitamin K antagonists**	1	0	NA
**Antihistaminica**	1	0	NA
**Antirheumatics**	1	0	NA
**Opioids**	1	0	NA

### Clinical measurements

Qualified healthcare professionals performed all adipometric examinations. Weight [kg] was determined in underwear and without shoes using a digital scale calibrated and standardized using a weight of known mass. Height [cm] was recorded using a stadiometer with participants standing on a flat surface at a right angle to the vertical board of the stadiometer. Abdominal circumference [cm] was measured with an inelastic measuring tape placed over the skin. While participants stood upright, circumference was taken at the level of the narrowest part of the torso as seen from the anterior view. Waist circumference (WC) [cm] was measured using a non-elastic tape at the midpoint between the last rib and the upper edge of the iliac crest at the end of the expiratory movement. The hip circumference (HC) [cm] was also measured using a non-elastic tape around the midline of the greater trochanter. BMI [kg/m^2^] was defined as body weight [kg] divided by the square of height [m^2^]. WHR was defined as WC / HC. Waist-to-height-ratio (WHtR) was defined as WC / height. All adipometric measurements were performed three times and the average was used for analysis.

### Cytokine measurement

After blood drawings, serum probes were immediately centrifuged at 3000 rpm for 10 min. The supernatant was aliquoted and stored in non-absorbing polypropylene tubes of 300 μl. Probes were shock-frozen in liquid nitrogen and stored in freezers at -80°C until further measurement. Cytokines were measured using the Bio-Plex Pro human cytokine Th1/Th2 immunoassay from Bio Rad, Germany, a 96-well kit that includes coupled magnetic beads and detection antibodies. This multiplex assay detects IL-2, IL-4, IL-5, IL-10, IL-12, IL-13, GM-CSF, IFN-γ and TNF-α.

### Actigraphy

In all but 5 participants (denied participation in this part) a 1-week actigraphy recording was performed, using the SenseWear Pro 3 actigraph (SWA; BodyMedia Inc.; Pittsburgh, Pennsylvania). This device is attached to the upper right arm and records 2-axis body acceleration, skin temperature, heat flux and galvanic skin response. Furthermore, the device detects epochs in which it is not worn (off-wrist periods). Recordings were performed between the telephone screening and assessment day. Participants kept a sleep and activity diary throughout the recording period. Actigraphic data was analyzed using SenseWear Professional Software Version 7 (BodyMedia Inc.) for estimation of number of steps and METs per 1-minute epoch using validated proprietary scoring algorithms included in the software. Several studies have demonstrated that the SWA device provides accurate estimates of energy expenditure during rest and activities of daily life, comparable to the gold standards of indirect calorimetry and doubly labeled water [27, 28]. The METs of an activity executed by an individual of a given body weight for a specified length of time were calculated by the equation:

$$MET=\frac{energy\text{​ }consumption\;[kcal]}{body\;weight\;[kg]\times time\;[h]}$$

Scored data was then imported into a customized Excel template with Visual Basic for Applications (VBA) macros (Microsoft Corp.), to organize data according to sleep-wake-periods, as follows: In the continuous data, ‘nighttime’ periods were detected based on the sleep scoring of the SenseWear software verified by sleep log data (i.e. ‘nighttime’ comprises the duration from first to last segment classified as sleep or laying down by the software which could be assigned to the nightsleep period according to the subjects sleep logs). However, ‘nighttime’ is not equivalent to ‘nightsleep’, as subjects could be awake or get up for a certain amount of time within the nighttime period). For daytime activity analyses, ‘days’ were not fixed to 0:00 a.m. to 11:59 p.m. but set from the middle of the preceding nighttime to the middle of the following nighttime. Therefore, ‘daytime’ was defined as the period between two successive nighttime periods. Only ‘days’ with a minimum recording duration of 20 hours and maximum off-wrist duration of 10% of the daytime period were evaluated. From the customized Excel-Template the variables steps (= sum of steps per 1min-segment within the daytime period) and METs (= average of METs per 1min-segment within the daytime period) were taken. The two variables were calculated for 7 weekdays and averaged to obtain mean values for 1 week. Datasets were included into analysis if they contained at least 5 days and nights of analyzable data (at least 3 week days/nights and both weekend days/nights). 140 participants fulfilled these requirements, comprising N = 80 (57.1%) obese patients and N = 60 (42.9%) non-obese controls.

### Statistics

Cytokine values were normalized using a logarithmic transformation. If cytokine levels were lower than the detection cut-off of the immunoassay, they were assigned a value of 0. Therefore, the following transformation formula was used: LN (cytokine value + 1). For all statistical analyses, the LN-transformed values were used.

Differences between study participants with and without adiposity were assessed by t-tests (age, BMI, WHR, weight, AC, WC, HC) following Gaussian distribution, and Chi^2^-tests (gender and smoking). For correlation analyses of cytokines and adipometrics and age, Pearson correlation coefficients were calculated. For comparisons of obese and non-obese subgroups regarding cytokine levels, multivariate analyses of variance with repeated measures (MANOVA) were conducted, with levels of different cytokines representing the dependent variable and the factor "group" (1 = obese, 0 = non-obese) being the independent variable. Main effects of the factor "group" as well as group x cytokine level interactions were investigated. Analogously, patients with central obesity versus without central obesity had been compared. For investigations of main effects, a post-hoc ANOVA, as well as a post-hoc ANCOVA with age as covariable, were applied with each cytokine as a dependent variable and the respective groups (obese vs. non-obese; with central obesity vs. without central obesity) as factors.

For cytokine differences between groups of low vs. high physical activity, ANOVAs and, in cases of associations between age or BMI and cytokines, steps or METs (as indicated by correlations |r| ≥ 0.3), ANCOVAs were calculated with age and/or BMI as covariable. Separation into groups of high and low physical activity and high and low METs was performed by median split of the respective variable within the total sample and the respective subgroups. The level of significance was set at p < 0.05 for all analyses.

## Results

200 participants, 117 obese and 83 non-obese, were included in the study and analyses. Socio-demographic differences between general obesity and non-obese groups are depicted in table 2. The full sample was also split into groups with central obesity (n = 69) and without central obesity (n = 131).

**Table 2:** Sociodemographic data and measures of obesity comparing obese and non-obese participants.

	Obese (n = 117)	Non-obese (n = 83)	p values
**Age [years] (mean ± SD)**	40.52 ± 13.26	34.20 ± 11.69	p = 0.001^a^
**Sex (female/male)**	77/40	49/34	p = 0.373^b^
**Smoker (yes/no)**	28/89	14/69	p = 0.291^b^
**BMI [kg/m** ^**2**^ **] (mean ± SD)**	44.28 ± 7.48	23.75 ± 3.02	p < 0.001^a^
**Weight [kg] (mean ± SD)**	128.74 ± 24.83	70.92 ± 12.70	p < 0.001^c^
**Abdominal circumference [cm] (mean ± SD)**	130.75 ± 18.11	85.01 ± 10.47	p < 0.001^c^
**Waist circumference [cm] (mean ± SD)**	121.65 ± 19.04	80.33 ± 10.68	p < 0.001^c^
**Hip circumference [cm] (mean ± SD)**	140.66 ± 16.15	101.07 ± 6.84	p < 0.001^c^
**Waist-to-hip-ratio (WHR) (mean ± SD)**	0.87 ± 0.10	0.79 ± 0.09	p < 0.001^c^
**Waist-to-hight-ratio (WHtR) (mean ± SD)**	0.71 ± 0.10	0.47 ± 0.05	p < 0.001^c^

^a^ independent t-test,

^b^ Chi^2^-test,

^c^ Mann-Whitney-U-test.

In the total sample, no cytokines showed correlations with age, whereas for IL-5, IL-10, IL-12 and IL-13 significant weak positive correlations with all adipometrics were found. L-5 generally showed the highest correlations (r > 0.30) when compared to the other cytokines. In the non-obese sample, significant correlations between cytokines and adipometrics were found for IL-5 and IL-10 only. In the obese sample, IL-10, IL-12 and IL-13 showed significant weak positive correlations with all adipometrics, however, IL-5 was only associated with waist circumference and WHtR. Also, IL-2, IL-4, IFN-γ and TNF-α exhibited significant but weak positive associations with age. All correlation coefficients are depicted in table 3.

**Table 3:** Pearson correlations between LN-transformed cytokine levels and age and adipometrics (correlations ≤ -0.3 and ≥ 0.3 are highlighted in bold font).

	Age [years]		BMI [kg/m^2^]		Weight [kg]		AC [cm]		WC [cm]		HC [cm]		WHR		WHtR	
Cytokines ^A^	r	P	r	p	r	p	r	p	r	p	R	p	r	p	r	p
**TOTAL SAMPLE**																
**IL-2 [pg/ml]**	0.126	0.075	0.143	0.044	0.152	0.032	0.127	0.082	0.155	0.028	0.127	0.074	0.115	0.105	0.146	0.040
**IL-4 [pg/ml]**	0.141	0.046	0.101	0.156	0.115	0.106	0.106	0.149	0.121	0.088	0.097	0.174	0.091	0.199	0.107	0.131
**IL-5 [pg/ml]**	0.115	0.104	**0.326**	<0.001	**0.339**	**<0.001**	**0.320**	**<0.001**	**0.352**	**<0.001**	**0.302**	**<0.001**	0.269	<0.001	**0.340**	**<0.001**
**IL-10 [pg/ml]**	0.102	0.150	0.244	<0.001	0.275	<0.001	0.291	<0.001	0.271	<0.001	0.222	0.002	0.218	0.002	0.246	<0.001
**IL-12 [pg/ml]**	0.062	0.381	0.284	<0.001	**0.302**	**<0.001**	0.277	<0.001	0.289	<0.001	0.248	<0.001	0.209	0.003	0.274	<0.001
**IL-13 [pg/ml]**	0.072	0.312	0.268	<0.001	0.294	<0.001	0.286	<0.001	0.296	<0.001	0.257	<0.001	0.215	0.002	0.276	<0.001
**GM-CSF [pg/ml]**	0.120	0.090	0.140	0.049	0.117	0.098	0.156	0.032	0.144	0.042	0.113	0.112	0.106	0.137	0.153	0.030
**IFN-γ [pg/ml]**	0.105	0.139	0.199	0.005	0.184	0.009	0.190	0.009	0.202	0.004	0.176	0.013	0.135	0.057	0.207	0.003
**TNF-α [pg/ml]**	0.152	0.032	0.176	0.013	0.192	0.006	0.171	0.019	0.200	0.005	0.170	0.016	0.145	0.041	0.185	0.009
**NON-OBESE SAMPLE**																
**IL-2 [pg/ml]**	-0.100	0.366	0.025	0.821	0.069	0.537	0.101	0.374	0.094	0.396	-0.094	0.397	0.169	0.126	0.068	0.542
**IL-4 [pg/ml]**	-0.098	0.376	0.046	0.679	0.101	0.364	0.096	0.399	0.091	0.411	-0.029	0.797	0.123	0.269	0.049	0.662
**IL-5 [pg/ml]**	-0.020	0.860	0.181	0.102	0.247	0.025	0.206	0.068	0.238	0.030	0.031	0.782	0.271	0.013	0.178	0.107
**IL-10 [pg/ml]**	-0.035	0.753	0.227	0.039	0.244	0.026	0.246	0.029	0.281	0.010	0.151	0.173	0.245	0.026	0.250	0.023
**IL-12 [pg/ml]**	-0.060	0.593	0.137	0.216	0.170	0.123	0.081	0.478	0.152	0.170	0.046	0.680	0.154	0.165	0.111	0.317
**IL-13 [pg/ml]**	-0.103	0.352	0.128	0.248	0.174	0.116	0.182	0.108	0.174	0.116	0.065	0.557	0.172	0.121	0.129	0.244
**GM-CSF [pg/ml]**	-0.078	0.486	0.063	0.573	0.105	0.345	0.114	0.319	0.095	0.394	0.024	0.829	0.093	0.401	0.057	0.608
**IFN-γ [pg/ml]**	-0.174	0.115	-0.010	0.927	0.072	0.519	0.062	0.588	0.052	0.639	0.012	0.917	0.051	0.647	-0.003	0.977
**TNF-α [pg/ml]**	-0.082	0.463	0.071	0.526	0.105	0.343	0.099	0.388	0.109	0.327	-0.041	0.713	0.156	0.160	0.079	0.475
**OBESE SAMPLE**																
**IL-2 [pg/ml]**	0.224	0.015	0.176	0.058	0.172	0.063	0.144	0.132	0.167	0.073	0.160	0.085	0.049	0.600	0.161	0.083
**IL-4 [pg/ml]**	0.265	0.004	0.125	0.179	0.129	0.166	0.145	0.131	0.141	0.130	0.129	0.167	0.049	0.599	0.131	0.158
**IL-5 [pg/ml]**	0.095	0.308	0.170	0.066	0.178	0.055	0.150	0.119	0.212	0.022	0.155	0.095	0.129	0.166	0.204	0.027
**IL-10 [pg/ml]**	0.145	0.118	0.248	0.007	0.291	0.001	0.265	0.005	0.247	0.007	0.199	0.032	0.126	0.175	0.210	0.023
**IL-12 [pg/ml]**	0.067	0.472	0.282	0.002	0.297	0.001	0.259	0.006	0.265	0.004	0.205	0.027	0.150	0.108	0.249	0.007
**IL-13 [pg/ml]**	0.127	0.171	0.232	0.012	0.272	0.003	0.236	0.013	0.271	0.003	0.219	0.018	0.143	0.123	0.243	0.008
**GM-CSF [pg/ml]**	0.192	0.038	0.149	0.108	0.076	0.413	0.134	0.164	0.135	0.148	0.089	0.340	0.074	0.427	0.173	0.062
**IFN-γ [pg/ml]**	0.233	0.011	0.108	0.247	0.051	0.585	0.073	0.449	0.110	0.238	0.045	0.626	0.089	0.338	0.143	0.125
**TNF-α [pg/ml]**	0.220	0.017	0.069	0.460	0.103	0.269	0.067	0.489	0.118	0.204	0.087	0.351	0.060	0.521	0.093	0.317

BMI = Body Mass Index. AC = Abdominal circumference. WC = waist circumference. HC = hip circumference. WHR = waist-to-hip-ratio. WHtR = Waist-to-height-ratio;

^A^ all cytokines transformed using formula LN(X+1).

Comparing cytokine levels between obese and non-obese groups, the overall MANOVA (with cytokines as repeated measures and group as factor) revealed a significant main effect of group (F = 7.189, p = 0.008) and a group*cytokine interaction (F = 2.553, p = 0.034). Post-hoc tests ANOVA (see table 4 left) revealed the obese group had significantly elevated serum concentrations of IL-5, IL-10, IL-12, IL-13, IFN-γ and TNF-α compared to the non-obese group. Following Bonferroni correction for multiple testing, differences remained significant for IL-5, IL-12 and IL-13.

**Table 4:** LN-transformed cytokine levels compared between general obesity and non-obese participants as determined by body-mass-index (BMI, left) and between central obesity and non-obese participants as determined by waist-hip ratio (WHR, right).

Cytokine ^A^	General Obesity determined by BMI				Central Obesity determined by WHR			
	Non-Obese (Mean ± SD)	Obese (Mean ± SD)	ANOVA ^B^ (p-value) ^D^	ANCOVA ^C^ (p-value) ^D^	Obese (Mean ± SD)	Non-Obese (Mean ± SD)	ANOVA ^B^ (p-value) ^D^	ANCOVA ^C^ (p-value) ^D^
**IL-2 [pg/ml]**	1.45 ± 0.99	1.64 ± 1.15	0.2399	0.4388	1.50 ± 1.07	1.69 ± 1.13	0.2428	0.4917
**IL-4 [pg/ml]**	1.45 ± 0.45	1.51 ± 0.48	0.4289	0.7455	1.46 ± 0.46	1.53 ± 0.48	0.2742	0.5850
**IL-5 [pg/ml]**	1.02 ± 0.57	1.36 ± 0.54	< 0.0001	0.0001	1.13 ± 0.55	1.38 ± 0.60	0.0033	0.0100
**IL-10 [pg/ml]**	1.13 ± 1.03	1.43 ± 0.86	0.0267	0.0539	1.17 ± 0.83	1.57 ± 1.10	0.0041	0.0104
**IL-12 [pg/ml]**	1.89 ± 0.99	2.28 ± 0.89	0.0047	0.0072	1.96 ± 0.84	2.41 ± 1.08	0.0013	0.0020
**IL-13 [pg/ml]**	1.48 ± 0.81	1.78 ± 0.61	0.0031	0.0053	1.56 ± 0.67	1.85 ± 0.77	0.0051	0.0089
**GM-CSF [pg/ml]**	3.34 ± 0.55	3.45 ± 0.70	0.2258	0.4055	3.34 ± 0.60	3.51 ± 0.72	0.0820	0.1902
**IFN- γ [pg/ml]**	4.53 ± 0.72	4.79 ± 0.62	0.0071	0.0161	4.60 ± 0.68	4.83 ± 0.65	0.0216	0.0507
**TNF-α [pg/ml]**	3.17 ± 0.58	3.39 ± 0.70	0.0194	0.0590	3.24 ± 0.61	3.40 ± 0.75	0.1043	0.2897

^A^ all cytokines transformed using formula LN(X+1),

^B^ ANOVA with group as factor,

^C^ ANCOVA with group as factor and age as covariate,

^D^ Bonferroni-Correction for Multiple Testing: α(0.05) → p = 0.0055; α(0.01) → p = 0.0011.

When comparing subjects with and without central obesity the overall MANOVA revealed a significant main effect of group (F = 7.125, p = 0.008) and group*cytokine interaction (F = 2.952, p = 0.016). In the post-hoc ANOVA (see table 4 right), subjects with central obesity showed significantly elevated serum concentrations of IL-5, IL-10, IL-12, IL-13 and IFN-γ compared to the group without central obesity (after Bonferroni correction, differences remained significant for all but IFN-γ).

In the subsample assessed for activity levels, correlations between cytokine levels and age or adipometrics were comparable to those found in the total sample (results not shown). Subjects were divided into groups of high vs. low activity, assessed by median split of average number of steps (activity) or METs (energy expenditure). Average number of steps significantly correlated with BMI in the total sample (r = -0.556) and the obese subsample (r = -0.450) as well as with age in obese (r = -0.306) and non-obese (r = 0.317) subsamples. Average METs correlated with BMI in the total sample (r = -0.803), as well as the obese (r = -0.589) and the non-obese subsample (r = -0.344); but for correlations with age r was below 0.30.

A MANOVA with cytokine levels as repeated measure and activity group (steps) as factor revealed a significant main effect of activity group (F = 8.224, p = 0.005) as well as a group*cytokine interaction (F = 3.077, p = 0.014). A post-hoc ANOVA (Table 5) showed a significant decrease in cytokine levels in the active group for IL-2, IL-10, IL-12, IL-13, IFN-γ and TNF-α, as well as a tendency for IL-4 and IL-5 (after Bonferroni correction, differences remained significant for IL-10 and IL-13). When activity groups were compared in the subsamples, effects were only found in the obese group. Here, the overall MANOVA again revealed a significant main effect of activity group (F = 8.428, p = 0.005) and a significant group*cytokine interaction (F = 3.096, p = 0.015) and the post-hoc ANOVA results showed decreased cytokine levels in the active obese compared to the inactive obese in all cytokines but GM-CSF and IFN-γ (Table 5). Again, after Bonferroni correction, only differences for IL-10 and IL-13 remained significant.

**Table 5:** Cytokine levels in subjects with high versus low physical activity determined by average number of steps during the wake-phase.

Physical Activity determined by average number of steps												
	Total Sample (N = 140)				Obese Sample (N = 80)				Non-Obese Sample (N = 60)			
	Low activity (≤ 8.600 steps)	High activity (> 8.600 steps)	ANOVA p-values ^B^	ANCOVA p-values ^C^	Low activity (≤ 6.800 steps)	High activity (> 6.800 steps)	ANOVA p-values ^B^	ANCOVA p-values ^D^	Low activity (≤ 11.000 steps)	High activity (> 11.000 steps)	ANOVA p-values ^B^	ANCOVA p-values ^E^
**Age**	40.99 ± 14.21	36.09 ± 12.10	0.0300	-	43.58 ± 13.78	40.43 ± 12.57	0.2887	-	30.70 ± 12.00	37.13 ± 11.88	0.0413	-
**BMI**	41.25 ± 11.22	29.14 ± 9.62	<0. 0001	-	47.06 ± 7.82	41.16 ± 6.78	0.0005	-	23.63 ± 2.93	23.00 ± 3.01	0.4210	-
**IL-2 [pg/ml]** ^**A**^	1.74 ± 1.11	1.34 ± 1.09	0.0324	0.2381	1.98 ± 1.07	1.29 ± 1.19	0.0078	0.0835	1.59 ± 1.04	1.25 ± 0.99	0.1951	0.2410
**IL-4 [pg/ml]** ^**A**^	1.55 ± 0.52	1.41 ± 0.43	0.0817	0.3463	1.66 ± 0.57	1.36 ± 0.43	0.0076	0.0480	1.43 ± 0.41	1.42 ± 0.50	0.8608	0.9803
**IL-5 [pg/ml]** ^**A**^	1.27 ± 0.63	1.06 ± 0.63	0.0519	0.9819	1.48 ± 0.54	1.18 ± 0.64	0.0298	0.1315	0.85 ± 0.65	1.06 ± 0.59	0.1822	0.1603
**IL-10 [pg/ml]** ^**A**^	1.55 ± 1.01	1.01 ± 0.91	0.0012	0.0272	1.71 ± 0.79	1.08 ± 0.89	0.0012	0.0118	1.18 ± 1.22	1.06 ± 0.99	0.6809	0.6736
**IL-12 [pg/ml]** ^**A**^	2.32 ± 1.00	1.85 ± 1.02	0.0062	0.1340	2.51 ± 0.86	1.96 ± 1.06	0.0130	0.0863	1.84 ± 1.19	1.94 ± 0.94	0.7162	0.6515
**IL-13 [pg/ml]** ^**A**^	1.85 ± 0.66	1.46 ± 0.77	0.0017	0.0514	1.98 ± 0.65	1.55 ± 0.61	0.0029	0.0254	1.58 ± 0.64	1.43 ± 0.97	0.4870	0.5814
**GM-CSF [pg/ml]** ^**A**^	3.48 ± 0.71	3.31 ± 0.56	0.1027	0.5645	3.57 ± 0.83	3.34 ± 0.53	0.1368	0.5224	3.29 ± 0.49	3.34 ± 0.60	0.7654	0.6935
**IFN- γ [pg/ml]** ^**A**^	4.79 ± 0.59	4.53 ± 0.80	0.0306	0.3705	4.89 ± 0.66	4.67 ± 0.62	0.1203	0.4063	4.61 ± 0.47	4.37 ± 0.97	0.2204	0.3512
**TNF-α [pg/ml]** ^**A**^	3.41 ± 0.64	3.16 ± 0.65	0.0206	0.2887	3.58 ± 0.68	3.20 ± 0.64	0.0125	0.0687	3.18 ± 0.57	3.12 ± 0.57	0.6876	0.7606

^A^ all cytokines transformed using formula LN(X+1),

^B^ ANOVA with activity group as factor,

^C^ ANCOVA with activity group as factor and BMI as covariate,

^D^ ANCOVA with activity group as factor and age & BMI as covariates,

^E^ ANCOVA with activity group as factor and age as covariate, Bonferroni-correction for Multiple Testing: α(0.05) → p = .0055; α(0.01) → p = .0011.

ANCOVAs with age and/or BMI as covariates revealed that most differences between groups depended on the BMI. In the overall sample, differences for IL-10 remained significant and trended for IL-13. Within the obese subsample, differences for IL-4, IL-10, IL-13 were significant and trended towards significance for IL-2, IL-12 and TNF-α (Table 5).

Regarding energy expenditure (METs), the overall MANOVA revealed a significant main effect of energy group (F = 4.117, p = 0.044). Post-hoc ANOVA results showed increased levels of IL-5, IL-10 and IL-13 in subjects with low energy expenditure in the total sample but not in the two subsamples. None of these differences remained significant after Bonferroni correction and none was significant after BMI was included as covariate (Supplement 1).

## Discussion

We performed a cross-sectional investigation of serum levels of nine different cytokines in 200 participants. To the authors’ knowledge, this is the first report on IL-2, IL-13 and GM-CSF levels in central obesity. Significant elevations of IL-5, IL-10, IL-12, IL-13, IFN-γ and TNF-α in generally obese compared to non-obese were found. Looking specifically at central obesity, we found higher levels of IL-5, IL-10, IL-12 and IL-13 and IFN-γ in participants with compared to those without central obesity. After Bonferroni-correction, IL-5, IL-12 and IL-13 were found to be elevated in both general and central obesity, and IL-10 elevated in the central obese. In accordance with our hypothesis, these results provide further evidence for the presence of a low-grade systemic inflammation in obesity, in which more than the commonly described adipokines IL-1, IL-6 and TNF-α are involved.

Several mechanisms have been identified as contributing to obesity-related inflammation: hyperplasic and hypertrophic adipocytes synthesize pro-inflammatory adipokines, macrophages migrate into the AT, where polarization from M2 to M1 macrophages is enhanced [11], whereas the T_H_2/ T_H_1 ratio and T_reg_ cell activity is reduced [10]. These processes are suggested to lead to a shift in cytokine levels from cytokines with anti-inflammatory properties to pro-inflammatory cytokines in obesity. Although in-vitro and animal studies favour cytokines secreted by M1 macrophage and T_H_1-cells to be up-regulated in obesity, our serum concentration data show that both M1 and M2 as well as T_H_1 and T_H_2-cytokines are likely affected in obesity and that not only pro-inflammatory, but also anti-inflammatory cytokines, such as IL-5, IL-13 and IL-10, show higher serum concentrations in obese participants compared to healthy controls. These findings are supported by other studies comparing subjects with MetS to healthy controls. In one study, M1 and M2 and T_H_1 and T_H_2-cytokines IFN-γ and IL-12 were significantly increased, as were the anti-inflammatory cytokines IL-4, IL-5 and IL-13 [18]. Overweight and obese subjects had higher levels of IL-12 than a normal-weight group, and this correlated with BMI and the grade of abdominal obesity [17]. Previous reports on IL-10 are heterogeneous with elevated levels in obese females [19], or reduced levels in obese subjects with either invariable concentrations in response to diet [16] or elevations associated with visceral fat loss [29]. In another study, IL-10 significantly increased, whereas TNF-α and IL-6 decreased following calorie restriction [30]. At the same time, anti-inflammatory IL-10 inhibited HFD-induced weight gain, IR and glucose intolerance [31].

As further hypothesized, though only in the obese group, the finding of pro- as well as anti-inflammatory cytokines being elevated in subjects with lower physical activity points towards a link between exercise and cytokine levels. However, when controlled for BMI, which was highly negatively correlated with both number of steps and METs, statistical significance was only reached in cytokines with anti-inflammatory properties, IL-4, IL-10 and IL-13, with trends in pro-inflammatory IL-2, IL-12 and TNF-α. Notably, the contrast between the significant differences for physical activity levels within the obese and virtually no difference within the non-obese, remained after allowing for covariates. These findings underline the impact of BMI on both physical activity and cytokine production. The higher BMI in the obese group with lower activity as well as the negative correlations between BMI, steps and METs also point towards this strong connection between BMI and physical activity that may depend on each other. The results may further indicate a generally higher production of both anti- and pro-inflammatory cytokines in obese participants with low activity, a pathophysiological relationship that may not occur in normal weight subjects without bloated fat cells and boosted secretion of cytokines. Congruent with these findings, the positive impact of physical activity on obesity, obesity-related diseases and inflammation has been substantiated by a large body of evidence [25, 32], though the influence of age, BMI, or other adipometrics may also account for some negative study results [33, 34]. The differences observed only within the obese, as well as the generally strong relationship to BMI, may relate to the finding that exercise and diet impact on most inflammatory mediators within AT but not within skeletal muscle [35]. Next to weight and body mass, physical activity may further mitigate inflammation by improving endothelial function, increasing insulin sensitivity, enhancing liver health and increasing tissue angiogenesis and blood flow [32, 36, 37]. Cytokine changes have also been observed in psychiatric disorders associated with disturbed wakefulness regulation and altered physical activity, such as mania and attention deficit hyperactivity disorder [38]. As a secondary finding, the discrepant findings of a positive correlation between number of steps and age within the non-obese but a negative correlation within the obese is worth noting and warrants further investigation. Concerning energy expenditure, findings of both significantly elevated levels of cytokines with notably anti-inflammatory properties IL-5, IL-10 and IL-13 within the uncontrolled total group again lost significance when controlled for BMI, a factor which appears more closely related to the amount of energy expenditure than the extent of cytokine production.

Concerning the device used to detect the number of steps and estimates of energy expenditure and the relatively low median METs observed in the study sample, it should be noted that mean resting energy expenditure estimated by the SWA was found to be approximately 70% of the total energy expenditure [39]. During low and moderate activity, as observed in the majority of participants in this study, the SWA was found to provide precise estimates [40], thus providing high validity for the results presented. However, limitations of the SWA need to be considered: the accuracies of estimates of energy expenditure are reported to depend on the type of activity [41–44]; for example, the SWA is not able to accurately detect some everyday activities like uphill walking, resulting in the risk of an underestimation of energy expenditure [41, 44]. Further, several studies described an under- or overestimation of energy expenditure by the SWA device when participants were obese or otherwise unhealthy [45–47].

The weak but consistently positive correlations between cytokines and adipometrics, predominately found in the obese group, and for those cytokines that differed significantly in group comparisons, suggest a relation between cytokine levels and the amount and distribution of visceral AT. This is consistent with findings showing altered cytokine secretion to depend on the amount of visceral rather than general obesity [20, 48]. The distribution of AT may be a factor in alterations of cytokine levels, and may also relate obesity to highly prevalent comorbidities such as IR, diabetes, atherosclerosis, sleep disturbances, and asthma. The high prevalence of these comorbidities in obese in contrast to non-obese subjects was also apparent in our study (Table 1).

Several studies suggest cytokines to be implicated in a common pathophysiology of obesity and these comorbidities. For example, in pre-diabetic obese females, IL-5, TNF-α and GM-CSF were elevated and correlated with hemoglobin A1c [49]. In contrast, deficiency of IL-5 resulted in increased adiposity and IR in animals [50]. The absence of TNF-α improves insulin receptor activity in muscle and fat tissue and reduces the amount of circulating fatty acids [51]. Accordingly, TNF-α is a stimulant for the release of free fatty acids from adipose tissue into blood circulation and is able to decrease insulin-modulating adiponectin [52]. Recently, IL-12 was also found linked to IR by showing gene expression of IL-12 family members in insulin-responsive tissue, such as skeletal muscle, liver, heart and white AT. The IL-12 family members were divergently regulated in relation to excessive nutrient intake, inflammatory stress and genetic obesity [53]. IFN-γ is known for its contribution to autoimmune pathologies such as lupus erythematosus, multiple sclerosis and insulin-dependent diabetes [54–56]. Growing evidence shows that there also is an autoimmune component in the pathogenesis of obesity-related diseases like type 2 diabetes [57, 58]. Thus, IFN-γ could be involved in the pathogenesis of obesity-linked type 2 diabetes and its progressive course. Accordingly, obese IFN-γ-knockout mice showed decreased adipocyte size, improved insulin-sensitivity, and a M2-shift in macrophages and cytokine profile [59]. GM-CSF-knockout mice had both ameliorated IR and pro-inflammatory cytokine profile [60]. For GM-CSF, evidence was given that it may influence energy homeostasis centrally but not peripherally, since food intake and body weight were found decreased in rodents after central GM-CSF administration [61]. GM-CSF is able to stimulate differentiation of tolerogenic dendritic cells supporting T_reg_ and secreting more IL-10. Consistent with this, treatment of prediabetic non-obese mice with GM-CSF prevented the development of autoimmune diabetes [62]. On the other hand, GM-CSF was found enhanced in obese persons with prediabetes in comparison to a non-prediabetic obese control group [49]. IL-4 and IL-13 are able to generate a M2-state and disrupting M2 activation leads to obesity and IR [63, 64]. Obesity is also a risk factor for asthma, both conditions sharing high comorbidity rates and asthma is more pronounced in asthmatic patients with than without obesity [65, 7]. Inflammation in adipose tissue is able to provoke airway inflammation in multiple ways [66, 67]. Representing heterogeneous entities, in the most phenotype of asthma, type 2 immune responses are up-regulated with elevated levels of IL-4, IL-5, IL-13 and GM-CSF [68–70], whereas also pro-inflammatory T_H_1 cytokines and adipokines have been found elevated [71]. In obesity-related asthma, as one of the different phenotypes in asthma, an absence of T_H_2 biomarkers is often described. Thereby, obese asthmatics are less responsive to corticosteroid therapy [72]. In contrast and in agreement with our results for IL-5 and IL-13, T_H_2 participation and more than one phenotype cluster in obese asthmatics have been postulated [73]. Therefore, further investigations are necessary to determine obese asthmatic biomarkers, essential for treatment decisions, particularly because the response to conventional therapies in “T_H_2 low” obese asthma is less effective.

In contrast to these potentially inflammation-derived connections between disorders, a substantial number of obese participants are not suffering from metabolism-related disorders, called metabolically healthy obese, who potentially represent a variant of obese phenotypes [74]. In these subjects, one may hypothesize predominant T_H_2-immunity with high levels of anti-inflammatory IL’s like IL-5, IL-10 and IL-13. The positive effect of physical activity on both obesity and its related diseases, as only observed to only impact on cytokine levels within the obese, may also lead to a down-regulation of cytokines within these subjects [23]. In future research, it might be promising to consider the role of anti-inflammatory cytokines such as IL-5, IL-10 and IL-13 in relation to physical activity in staying metabolically healthy despite the presence of obesity.

A number of limitations of the present work need to be addressed. Our findings do not indicate whether cytokines are a cause or a consequence of obesity, nor the underlying molecular mechanisms. Groups differed in mean age, and though this was controlled for in the statistical analyses, matching for sex and age could increase power. Within the activity groups the BMI needed to be included as a mandatory covariate. In future investigations homogenous sociodemographies and adipometrics between groups could best help to further elucidate connections between cytokines and physical activity. The number of comorbidities differed between the two groups and may have influenced cytokine levels. The SWA device only estimates METs and steps and the algorithms used for calculations are proprietary and not fully open to scrutiny. The estimation of METs may not be accurate for the obese where energy expenditure in relation to body weight, given a greater proportion of bio-inactive fat mass, is lower than for normal weight [39]. Therefore, it would be desirable to develop obesity-specific algorithms for the SWA, which is otherwise a reliable instrument to assess a broader range of routine daily activity than other existing devices, like accelerometers or pedometers [75, 39].[6,69]

## Conclusion

We found differences in pro- as well as anti-inflammatory cytokines when comparing 117 obese and 83 non-obese participants. Among the tested cytokines, IL-5, IL-10, IL-12, IL-13 and IFN-γ were elevated in both general and central obesity. Physical activity modulated cytokine production in the obese, which may have a beneficial effect in prevention of comorbidities in obese subjects. Of the cytokines studied, IL-5 concentrations showed the strongest correlation with adipometrics and may be a possible research and drug target in obesity. Changes in cytokine concentrations may contribute to the development of secondary diseases of obesity and may be a promising drug target in these disorders.

## Supporting Information

S1 Table Cytokine levels in subjects with high versus low physical activity determined by average energy expenditure during the wake-phase. Legend: ^A^ all cytokines transformed using formula LN(X+1), ^B^ ANOVA with energy group as factor, ^C^ ANCOVA with energy group as factor and BMI as covariate, Bonferroni-correction for Multiple Testing: α(0.05) → p = .0055; α(0.01) → p = .001.
(PDF)
