# Supplementary material for: Inflammatory Cytokines in General and Central Obesity and Modulating Effects of Physical Activity
Source: PLoS One. 2015 Mar 17;10(3):e0121971. doi: 10.1371/journal.pone.0121971 (PMC4363366; doi:10.1371/journal.pone.0121971)
Supplement: S1 Table — Legend: A all cytokines transformed using formula LN(X+1), B ANOVA with energy group as factor, C ANCOVA with energy group as factor and BMI as covariate, Bonferroni-correction for Multiple Testing: α(0.05) → p = .0055; α(0.01) → p = .001. (PDF) [file pone.0121971.s001.pdf]

Supporting Information 1: Cytokine levels in subjects with high versus low physical activity determined by average energy expenditure during the wake-phase.

Physical Activity determined by average METs												
	Total Sample (N = 140)				Obese Sample (N = 80)				Non-Obese Sample (N = 60)			
	Low energy (< 1.500 METs)	High energy (≥ 1.500 METs)	ANOVA p-values [B]	ANCOVA p-values [C]	Low energy (< 1.200 METs)	High energy (≥ 1.200 METs)	ANOVA p-values [B]	ANCOVA p-values [C]	Low energy (≤ 1.951 METs)	High energy (> 1.951 METs)	ANOVA p-values [B]	ANCOVA p-values [C]
**Age**	42.73 ± 12.94	34.34 ± 12.55	0.0002	-	43.90 ± 13.92	40.10 ± 12.32	0.1998	-	33.77 ± 13.16	34.07 ± 11.55	0.9255	-
**BMI**	44.86 ± 8.00	25.54 ± 6.30	<0.0001	-	47.36 ± 7.07	40.86 ± 7.29	0.0001	-	24.21 ± 3.06	22.41 ± 2.60	0.0172	-
**IL-2 [pg/ml] [A]**	1.66 ± 1.19	1.43 ± 1.03	0.2042	0.3736	1.83 ± 1.18	1.44 ± 1.15	0.1349	0.6276	1.49 ± 1.10	1.36 ± 0.95	0.6143	0.5433
**IL-4 [pg/ml] [A]**	1.53 ± 0.52	1.42 ± 0.43	0.1947	0.7540	1.57 ± 0.57	1.46 ± 0.42	0.3253	0.8187	1.48 ± 0.46	1.38 ± 0.44	0.4083	0.4071
**IL-5 [pg/ml] [A]**	1.31 ± 0.62	1.02 ± 0.63	0.0064	0.4590	1.37 ± 0.62	1.29 ± 0.61	0.5530	0.8604	0.93 ± 0.64	0.98 ± 0.62	0.7426	0.5687
**IL-10 [pg/ml] [A]**	1.45 ± 0.93	1.10 ± 1.04	0.0418	0.8473	1.57 ± 0.96	1.22 ± 0.81	0.0856	0.3740	1.22 ± 1.23	1.02 ± 0.98	0.5069	0.8909
**IL-12 [pg/ml] [A]**	2.22 ± 1.02	1.95 ± 1.04	0.1276	0.1740	2.42 ± 0.92	2.06 ± 1.04	0.1094	0.4566	1.94 ± 1.18	1.83 ± 0.94	0.6968	0.8646
**IL-13 [pg/ml] [A]**	1.79 ± 0.66	1.52 ± 0.80	0.0312	0.7325	1.88 ± 0.67	1.65 ± 0.65	0.1251	0.4841	1.59 ± 0.79	1.41 ± 0.85	0.3905	0.5654
**GM-CSF [pg/ml] [A]**	3.46 ± 0.72	3.33 ± 0.54	0.2606	0.2402	3.48 ± 0.82	3.43 ± 0.56	0.7687	0.5301	3.37 ± 0.53	3.26 ± 0.55	0.4001	0.4573
**IFN- γ [pg/ml] [A]**	4.77 ± 0.63	4.55 ± 0.77	0.0660	0.5020	4.83 ± 0.67	4.74 ± 0.63	0.5383	0.8965	4.61 ± 0.51	4.37 ± 0.95	0.2249	0.1703
**TNF-α [pg/ml] [A]**	3.38 ± 0.67	3.20 ± 0.65	0.0968	0.3160	3.50 ± 0.72	3.28 ± 0.64	0.1683	0.4919	3.17 ± 0.61	3.13 ± 0.56	0.7674	0.7481

Legend: [A] all cytokines transformed using formula LN(X+1), [B] ANOVA with energy group as factor, [C] ANCOVA with energy group as factor and BMI as covariate

Bonferroni-correction for Multiple Testing: α(0.05) → p = .0055; α(0.01) → p = .0011
